# How physical activity affects young adults’ subjective wellbeing: the mediation role of subjective socioeconomic status and social support

**DOI:** 10.3389/fpsyg.2026.1703473

**Published:** 2026-06-03

**Authors:** Fangyan Lv, Run Feng, Dongzhe Shi, Dingguo Gao

**Affiliations:** 1 School of Marxism, Sun Yat-sen University, Guangzhou, China; 2 Department of Psychology, and Guangdong Provincial Key Laboratory of Social Cognitive Neuroscience and Mental Health, Sun Yat-sen University, Guangzhou, China; 3 Department of Sports Science and Physical Education, Guangzhou Xinhua University, Guangzhou, China

**Keywords:** physical activity, social support, subjective socioeconomic status, subjective wellbeing, young adults

## Abstract

**Background:**

While theoretical frameworks and empirical evidence consistently identify physical exercise as a key factor in promoting subjective wellbeing among young adults, the specific pathways and mechanisms underlying this relationship remain largely underexplored. This research aims to investigate the potential mechanism with subjective socioeconomic status and social support as mediators among Chinese young adults.

**Methods:**

This research utilized a cross-sectional approach in the study design to conduct an online survey among Chinese college students. Through convenience sampling, a total of 630 young adults (*M* = 19.61, SD = 1.13), aged 18 to 25, from two universities in Guangzhou, Guangdong Province, participated in this study and completed the questionnaires for physical activity, subjective wellbeing, subjective socioeconomic status, and social support. Data analysis for this study was performed utilizing the statistical software packages SPSS version 23.0 and PROCESS version 3.3. Correlation analysis and mediational analysis were employed in this study.

**Results:**

Results indicated that physical activity, subjective socioeconomic status, social support, and subjective wellbeing were significantly associated with each other. (1) Physical activity was significantly associated with subjective wellbeing (*r* = 0.27, *p* < 0.001), and physical activity directly predicted subjective wellbeing (*β* = 0.25, *t* = 6.18, *p* < 0.001). (2) Physical activity positively predicted subjective socioeconomic status (*β* = 0.10, *t* = 4.21, *p* < 0.001) and social support (*β* = 0.16, *t* = 4.05, *p* < 0.001), subjective socioeconomic status significantly positively predicted subjective wellbeing (*β* = 0.12, *t* = 3.39, *p* < 0.001) and social support (*β* = 0.17, *t* = 4.50, *p* < 0.001), social support significantly and positively predicted subjective wellbeing (*β* = 0.35, *t* = 9.56, *p* < 0.001). (3) Subjective socioeconomic status and social support played a chain mediating role between physical activity and subjective wellbeing.

**Conclusion:**

Physical activity could positively predict Chinese young adults’ subjective wellbeing through subjective socioeconomic status and social support as mediators, which indicates that young adults should regularly participate in physical activities and increase social support to enhance their mental health and overall sense of wellbeing.

## 1 Introduction

Physical activity is defined as any bodily movement produced by skeletal muscles that requires energy expenditure (Caspersen et al., 1985). As a modifiable health behavior, it is a well-documented determinant of physical, mental, and social wellbeing (Biddle et al., 2019; Warburton and Bredin, 2017; WHO, 2014; Wood et al., 2022). To optimize these health benefits, guidelines recommend regular moderate-to-vigorous exercise, muscle-strengthening activities, and reduced sedentary behavior (Bull et al., 2020; Rhodes et al., 2017; Zhang et al., 2022). However, despite these established benefits, global epidemiological data indicate that a significant proportion of adults remain insufficiently active. Specifically, adherence to recommended physical activity levels varies across regions, with a considerable proportion of adults failing to meet the thresholds—for instance, 43% in North America and 36.8% in Europe, as supported by epidemiological data (Ozemek et al., 2019).

Young adulthood is a critical developmental stage characterized by substantial physiological, psychological, and social transitions, and promoting physical activity during this period can produce long-term benefits (McKercher et al., 2014). Physical activity reduces obesity and cardiovascular disease risk (Elagizi et al., 2020; Holtermann et al., 2021; Wareham et al., 2005) and improves mental health, academic performance, and overall wellbeing (Corder et al., 2019; Haverkamp et al., 2020). Notably, subjective wellbeing among young students has been declining (Esposito et al., 2022; Grajek and Sobczyk, 2021; Reyes-Molina et al., 2022), threatening mental health, social integration, and future development. As an important self-care and health-promoting factor, physical activity is therefore essential for understanding and improving young adults’ wellbeing (Biddle et al., 2019; Fancourt et al., 2021; Penedo and Dahn, 2005; Warburton and Bredin, 2017; Rodríguez-Romo et al., 2022; Wood et al., 2022).

Subjective wellbeing is a multifaceted construct including life satisfaction, positive emotions, and low negative emotions (Diener et al., 1999). It predicts better physical health and longevity, making its enhancement an important public health goal (Diener and Chan, 2011). A large body of research supports the positive link between physical activity and subjective wellbeing (Buecker et al., 2021; Herbert et al., 2020; Liao C. et al., 2023; Liao T. et al., 2023; Wood et al., 2022), as emphasized in WHO guidelines. However, the underlying mechanisms remain unclear. Most studies focus on individual or biological factors, while the mediating roles of key social–psychological variables, especially subjective socioeconomic status (SSS) and social support, remain understudied among young adults, limiting the design of targeted interventions.

Physical activity exerts broad benefits for mental health and wellbeing (Biddle, 2016; Buecker et al., 2021; Chekroud et al., 2018; Hills et al., 2015; Mammen and Faulkner, 2013; An et al., 2020; Granero-Jiménez et al., 2022; Iwon et al., 2021; Lyulina et al., 2023; Pengpid and Peltzer, 2019). Meta-analyses confirm its effects in reducing depression and anxiety (Rebar et al., 2015; McMahon et al., 2017; Biddle, 2016; Chekroud et al., 2018; Stubbs et al., 2017). Longitudinal studies show that regular exercise rapidly improves life satisfaction and positive affect within 4 weeks (Iwon et al., 2021). Cross-national and large-scale studies further demonstrate that moderate-to-vigorous physical activity is associated with higher subjective wellbeing in young adults (Pengpid and Peltzer, 2019; An et al., 2020; Granero-Jiménez et al., 2022; Lyulina et al., 2023). Although prior research has examined mediators such as self-efficacy and neurobiological changes (Zhang and Chen, 2019), the roles of SSS and social support remain unclear. We therefore propose Hypothesis 1: Physical activity is positively associated with young adults’ subjective wellbeing.

Socioeconomic status includes both objective indicators and subjective perceptions of social hierarchy. SSS predicts health and well-being more strongly than objective socioeconomic status in many cases (Fors Connolly et al., 2018; Niu et al., 2023; Cundiff and Matthews, 2017) and is commonly measured using the MacArthur Scale (Adler et al., 2000; Euteneuer, 2014; Cundiff et al., 2017). Subjective socioeconomic status is primarily assessed using the widely recognized MacArthur Scale. As a single-item measurement tool, it employs a 10-rung ladder as a metaphor, allowing respondents to compare themselves with others and position themselves based on their perceived social hierarchy (Adler et al., 2000; Euteneuer, 2014), before selecting the rung that best represents their own social standing (Cundiff and Matthews, 2017). SSS is shaped not only by objective conditions but also by psychological characteristics (Euteneuer, 2014; Hoebel et al., 2015; Zhou et al., 2021).

According to Social Comparison Theory (Festinger, 1957), which serves as the primary theoretical foundation of this study, individuals evaluate their socioeconomic status by comparing themselves with others. According to Social Comparison Theory, individuals evaluate their social standing through self–other comparisons, and higher perceived social status predicts better wellbeing. Physical activity may enhance self-efficacy, social participation, and perceived competence, thereby improving subjective socioeconomic status. In turn, individuals with higher subjective socioeconomic status tend to have broader social networks and greater access to social support, which further promotes subjective wellbeing. This process is a key determinant of health across cultures (Nobles et al., 2013), and SSS often outperforms objective indicators in predicting health outcomes (Euteneuer et al., 2021; Hoebel and Lampert, 2020).

A growing literature confirms that SSS is a critical psychological predictor of young adults’ subjective wellbeing (Andersson, 2018; Euteneuer, 2014; Quansah et al., 2023). For example, Wang et al. (2023) found a strong association between SSS and wellbeing in a large Chinese sample. Stress theory explains this link: individuals with higher SSS enjoy better resources, greater resilience, and fewer stressful life events, all of which support wellbeing (George, 2010; Simone and Haas, 2013; Zhao et al., 2021).

Previous research has identified a correlation between physical activity and SSS (Stalsberg and Pedersen, 2010; Chen et al., 2015; Grzywacz and Marks, 2001; Lee et al., 2007). Among children and adolescents, SSS is positively related to physical activity participation (Ke et al., 2022). In Chinese adults, physical activity is related to socioeconomic indicators, especially education (Beenackers et al., 2012; Cleland et al., 2012). Socially patterned differences exist: middle-SES individuals more often engage in high-intensity occupational activity, lower-SES individuals in household labor, and higher-SES individuals in exercise participation (Chen et al., 2015). Similar patterns appear in high-income countries (Johnstone et al., 2019; Scottish Government, 2017; Stalsberg and Pedersen, 2010).

Despite these findings, the mediating role of SSS in the physical activity–wellbeing relationship remains largely unexplored among young adults. Although some studies have tested SSS as a mediator (Chen and Zhu, 2021), its function in the context of physical activity remains unclear. To address this gap, we propose Hypothesis 2: SSS mediates the relationship between physical activity and subjective wellbeing.

To further clarify the mechanism, this study introduces social support as a second mediator. Social support reflects the quality and function of social bonds and has been widely studied in mental health and wellbeing (Cobo-Rendón et al., 2020; Gençöz et al., 2004; Gülaçtı, 2010; Shin and Park, 2022; Siedlecki et al., 2014; Turner, 1981; Winefield et al., 1992; Yıldırım & Tanrıverdi, 2021, Yildirim et al., 2023; Schwarzer et al., 2004). Physical activity provides social interaction and is consistently associated with higher social support (Laird et al., 2016; Lindsay Smith et al., 2017; Mendonça et al., 2014; Swed et al., 2023; Treiber et al., 1991). Social support, in turn, buffers stress and promotes wellbeing. Notably, a study focusing on Chinese adolescents has identified social support as having a mediated effect on the association between subjective family socioeconomic status and life satisfaction (Liao C. et al., 2023; Liao T. et al., 2023).

Physical activity, social support, and subjective wellbeing are closely linked. Greater physical activity and social support predict higher wellbeing in young adults (Liao C. et al., 2023; Liao T. et al., 2023). Social support obtained through physical activity may partially explain its benefits for wellbeing. We therefore propose Hypothesis 3: Social support mediates the relationship between physical activity and subjective wellbeing.

**Figure 1:**
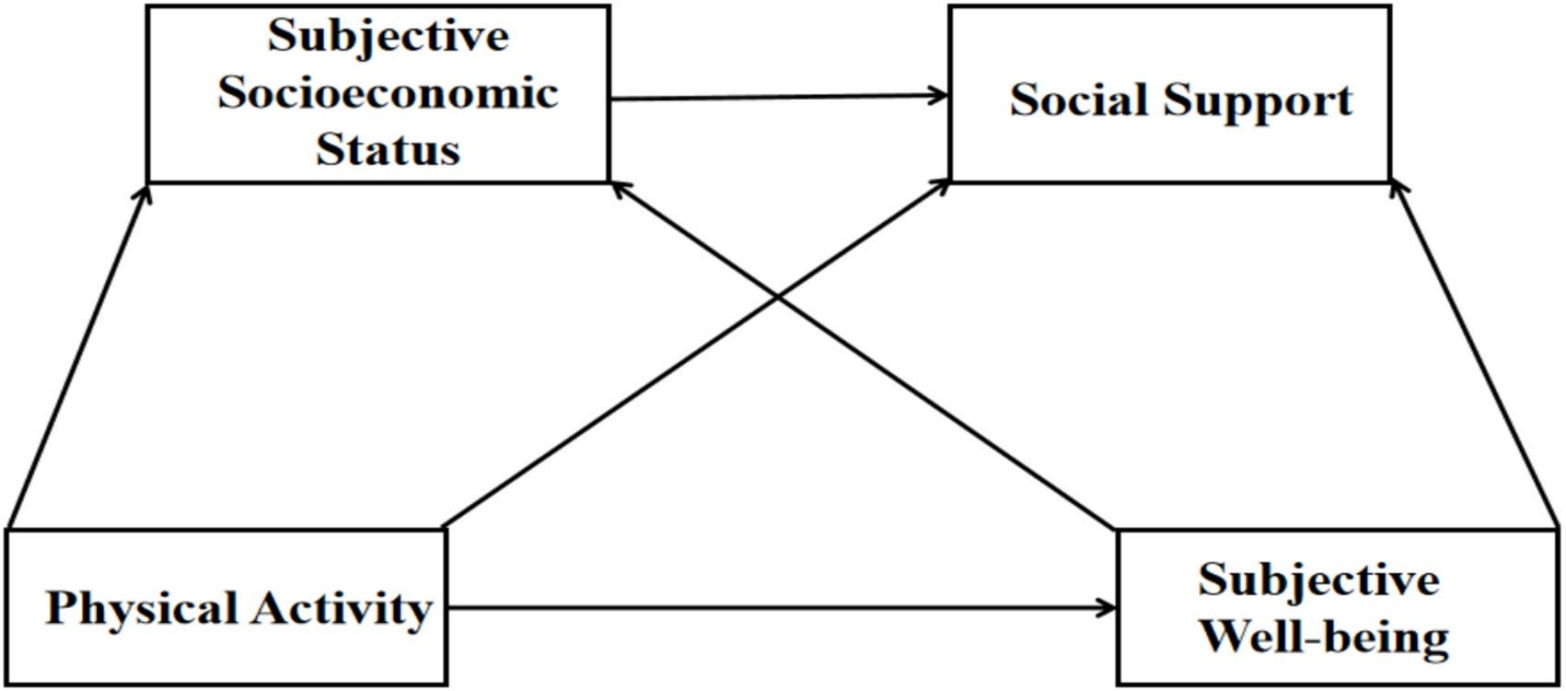
The proposed chain mediation model.

Understanding how physical activity, SSS, and social support jointly influence wellbeing can inform intervention strategies. Previous studies have shown that SES is related to the type and frequency of physical activity (Chen J. et al., 2022; Chen Y. et al., 2022; Shen et al., 2022). Integrating these lines of evidence, we propose that SSS and social support may function in a sequential pathway. Accordingly, Hypothesis 4: SSS and social support play a sequential chain mediating role between physical activity and subjective wellbeing (see Figure 1).

Given the cross-sectional design, we emphasize that the present model reflects statistical predictive pathways and correlational relationships rather than strict causal inference. Reverse causation is also possible: individuals with higher subjective wellbeing or stronger social support may be more likely to engage in physical activity.

Consistent with the social ecological model as a supplementary framework, individual behavior (physical activity), interpersonal resources (social support), and social status perception (subjective socioeconomic status) interact to shape subjective wellbeing. In summary, this study develops and tests a serial mediation model to examine how SSS and social support help explain the association between physical activity and subjective wellbeing among young adults. We hypothesize that:

1. Physical activity is positively associated with subjective wellbeing;
2. SSS mediates the physical activity–wellbeing link;
3. Social support mediates the physical activity–wellbeing link;
4. SSS and social support function as a sequential chain mediator.

## 2 Methods

### 2.1 Participants and procedure

Given the constraints of time and resources, a total of 630 young adults were recruited via convenience sampling from two universities in Guangzhou, Guangdong Province, China. These institutions were selected based on geographical proximity and accessibility, which facilitated efficient data collection. To ensure the homogeneity of the sample and the validity of the results regarding physical activity and subjective wellbeing, strict inclusion and exclusion criteria were applied. Participants were included if they were full-time students aged 18 to 24 years. Exclusion criteria comprised individuals with physical disabilities or chronic health conditions that would limit their ability to engage in physical activity, as well as those with a history of severe mental illness. Exclusions were determined based on self-reported health status in the online questionnaire, given the anonymous survey design. Ultimately, the sample consisted of 418 females and 212 males, aged 18 to 24 years (*M* = 19.61, SD = 1.13). All data were collected using Wenjuanxing, a professional online survey platform in China. At the outset of the survey, informed consent was secured from the participants, with the understanding clearly communicated that they had the option to discontinue their participation at any juncture. This could be achieved simply by closing their web browser, ensuring that their responses would not be recorded. All participants voluntarily and anonymously took part in this survey. The completion of the questionnaire was conducted at the participant’s own pace, with forced progression logic applied to ensure that participants could advance to the following page only after fully completing the preceding one. This study received ethical approval from the Research Ethics Committee at the first author’s affiliated university.

### 2.2 Measures

#### 2.2.1 Physical activity

In this study, the revised Physical Activity Rating Scale (PARS-3; Liang, 1994), which is a 3-item self-reported scale comprising intensity, duration, and frequency, was employed to assess physical exercise. Comprising three items, the PARS-3 evaluates exercise intensity, frequency, and single exercise duration, with each item categorized into five levels and scored on a 1–5 scale. Prior studies have shown the strong applicability of this scale in a large sample of Chinese college students (Wang et al., 2022; Yang et al., 2021). In this study, participants’ actual physical activity scores fell within the 0 to 100 range. The scale demonstrated relatively high test–retest reliability, with a Cronbach’s *α* coefficient of 0.82. Notably, as a brief self-report instrument, the PARS-3 cannot fully capture the complexity, type, context, or real-time intensity of physical activity, which represents an inherent limitation of the study design.

#### 2.2.2 Subjective wellbeing

The Subjective Wellbeing Scale (SWS) was utilized to comprehensively evaluate participants’ overall quality of life, encompassing both cognitive and emotional dimensions through its two integral subscales: the Satisfaction with Life Scale (SWLS) and the Positive and Negative Affect Scale (PANAS).

The SWLS, originally developed by Diener et al. (1985) and subsequently revised by Leung and Leung (1992), served as the instrument to measure life satisfaction. Focusing on the cognitive aspect of subjective wellbeing, this five-item scale adopted a 7-point Likert response format, ranging from “completely disagree” to “completely agree.” Life satisfaction scores on this scale could range from 5 to 35, with higher values indicating a greater degree of life satisfaction. In the present study, participants’ scores spanned the entire range, and the scale demonstrated excellent internal consistency, with a Cronbach’s α reliability coefficient of 0.85.

To assess the emotional component of wellbeing, the PANAS was employed [developed by Watson et al. (1988) and revised by Huang et al. (2003)]. This scale was composed of two subscales: Positive Affect (PA) and Negative Affect (NA). Each subscale contained 10 adjective items that described positive or negative emotions, resulting in a total of 20 items. Responses were scored on a 5-point Likert scale, from 1 (“nothing at all”) to 5 (“very strong”). The total scores for the PA and NA subscales ranged from 10 to 50, where higher scores indicated more intense experiences of positive or negative emotions. The Cronbach’s *α* coefficients for the PA and NA subscales were 0.82 and 0.84, respectively, and the overall α coefficient was 0.84, indicating satisfactory internal consistency in the present sample. The reliability and validity of the PANAS have been well-established in previous studies among Chinese populations.

#### 2.2.3 Subjective socioeconomic status

The MacArthur Scale of Subjective Socioeconomic status, a widely validated instrument, employs a 10-rung ladder metaphor to assess individuals’ perceived social standing (Adler et al., 2000). This scale has demonstrated particular utility in evaluating Chinese rural–urban migrants, with research confirming its reliability and cross-cultural validity (Huang et al., 2017). The higher ladder positions reflect greater perceived social standing. Participants self-locate on the ladder by comparing their status to peers, family, and colleagues, selecting the rung that best represents their social position. Scoring ranges from 1 (“lowest subjective socioeconomic status”) to 10 (“highest subjective socioeconomic status”), with prior studies in diverse populations—including Chinese adults (Chen et al., 2020) and Italian adolescents (Giatti et al., 2012)—validating its psychometric properties. This intuitive measure has become a standard tool for capturing nuanced perceptions of social hierarchy in behavioral research.

#### 2.2.4 Social support

The Social Support Rating Scale (SSRS), developed by Xiao (1999), is a 10-item self-report instrument designed to measure three distinct components of social support: subjective support, objective support, and support utilization. The scale has demonstrated robust psychometric properties in studies of Chinese college student populations. Subjective Support (4 items): Assesses emotional perceptions of respect, support, and understanding. Objective Support (3 items): Measures tangible forms of received support. Support Utilization (3 items): Evaluates the degree to which individuals engage with available social resources. Responses are scored using a standardized metric. Prior research has reported a Cronbach’s α reliability coefficient of 0.61 for this three-factor model, which is considered acceptable for exploratory studies in social science contexts (Xiao, 1999). This finding aligns with the instrument’s design, which prioritizes contextual validity in measuring multi-dimensional social support structures. The validated tool enables systematic assessment of social support networks and their functional impact on wellbeing, providing a robust framework for analyzing interpersonal resources in health-related research.

### 2.3 Data analysis

In this study, data integrity was robust, with minimal missing values across participants, obviating the need for data deletion. Statistical analyses were executed using SPSS 23.0, with statistical significance set at a two-tailed *p* < 0.05 threshold. The analytical process commenced with an assessment of common method bias, followed by the computation of descriptive statistics and bivariate correlations among study variables. Building on these preliminary findings and the established hypotheses, a linear regression model was constructed to examine the relationship between physical activity and subjective wellbeing. Leveraging PROCESS 3.3 for SPSS (Hayes, 2018), Model 6 was specifically chosen due to its ability to evaluate the sequential mediation pathways involving subjective socioeconomic status and social support (Figure 1). Age and gender were included as control variables to account for potential confounding effects. To rigorously test the proposed mediation mechanisms, a non-parametric Bootstrap procedure with 5,000 resamples was employed. Mediation effects were deemed significant when the 95% confidence interval did not include zero, providing robust evidence for the hypothesized relationships. This analytical approach ensures both methodological rigor and interpretability of the results.

Before testing the mediation model, multicollinearity diagnostics were conducted using Tolerance and VIF values. The analysis revealed that all Tolerance values were greater than 0.80 and all VIF values were less than 2.40. As these values are well within the acceptable range (Tolerance > 0.10; VIF < 10.00), we confirmed that multicollinearity did not pose a threat to the validity of the model or the stability of the indirect effect estimates.

## 3 Results

### 3.1 Common method bias test

We employed Harman’s one-factor test to examine whether common method bias exists in the data. During the test, we utilized the factor analysis function in SPSS to extract the first eigenvalue from the data matrix. The test results showed that the first eigenvalue explained 19.04% of the total variance, well below the 40% threshold, which is typically regarded as representing the majority of explained total variance. Therefore, based on Harman’s one-factor test, common method bias is unlikely to have biased the present study results (Zhou and Long, 2004). These results indicate that there is no significant common method bias in the study data.

### 3.2 The relationship between physical activity, subjective wellbeing, subjective socioeconomic status, and social support

To begin with, we conducted an independent samples *t*-test to explore possible disparities in gender across the variables delineated in Table 1. This analysis revealed significant differences (*p* < 0.05) between male and female students in terms of physical activity and subjective wellbeing. Subsequently, Table 2 shows the findings from the descriptive statistics and bivariate correlations about the key variables under examination.

**Table 1:** Gender differences in variables.

Variable	Gender (*M* ± SD)		*t*
	Females (*n* = 418)	Males (*n* = 212)	
Physical activity	15.39 ± 15.08	26.84 ± 26.85	6.849^***^
Subjective wellbeing	47.30 ± 10.37	50.54 ± 11.85	3.516^***^
Subjective socioeconomic status	5.31 ± 1.58	5.50 ± 1.92	1.270
Social support	35.98 ± 5.34	35.30 ± 6.16	−1.430

*p < 0.05, **p < 0.01, and ***p < 0.001.

**Table 2:** Descriptive statistics and correlation analysis of variables.

Variable	M ± SD	1	2	3	4
Physical activity	19.37 ± 20.53	_			
Subjective wellbeing	48.40 ± 10.99	0.27^***^	_		
Subjective socioeconomic status	5.375 ± 1.70	0.11^**^	0.21^***^	_	
Social support	35.75 ± 5.63	0.15^***^	0.39^***^	0.19^***^	_

*p < 0.05, **p < 0.01, and ***p < 0.001.

As illustrated in Table 2, significant correlations were observed among the primary variables. Specifically, physical activity was significantly positively associated with subjective wellbeing, thereby supporting our first hypothesis that heightened physical activity is positively correlated to greater subjective wellbeing in adulthood. Moreover, elevated levels of physical activity were also significantly positively associated with higher subjective socioeconomic status and increased social support. Notably, the correlation between subjective socioeconomic status and social support was positive, indicating that young adults with higher subjective socioeconomic status tend to report greater social support. Additionally, the significant positive correlation between subjective wellbeing and social support suggests that enhanced social support is correlated with higher subjective wellbeing. Furthermore, considering the variable of gender demonstrates varying levels of correlation with the factors under scrutiny, it will be integrated as a control variable in the forthcoming analyses.

### 3.3 Mediated model: the effects of subjective socioeconomic status and social support on the relationship between physical activity and subjective wellbeing

**Figure 2:**
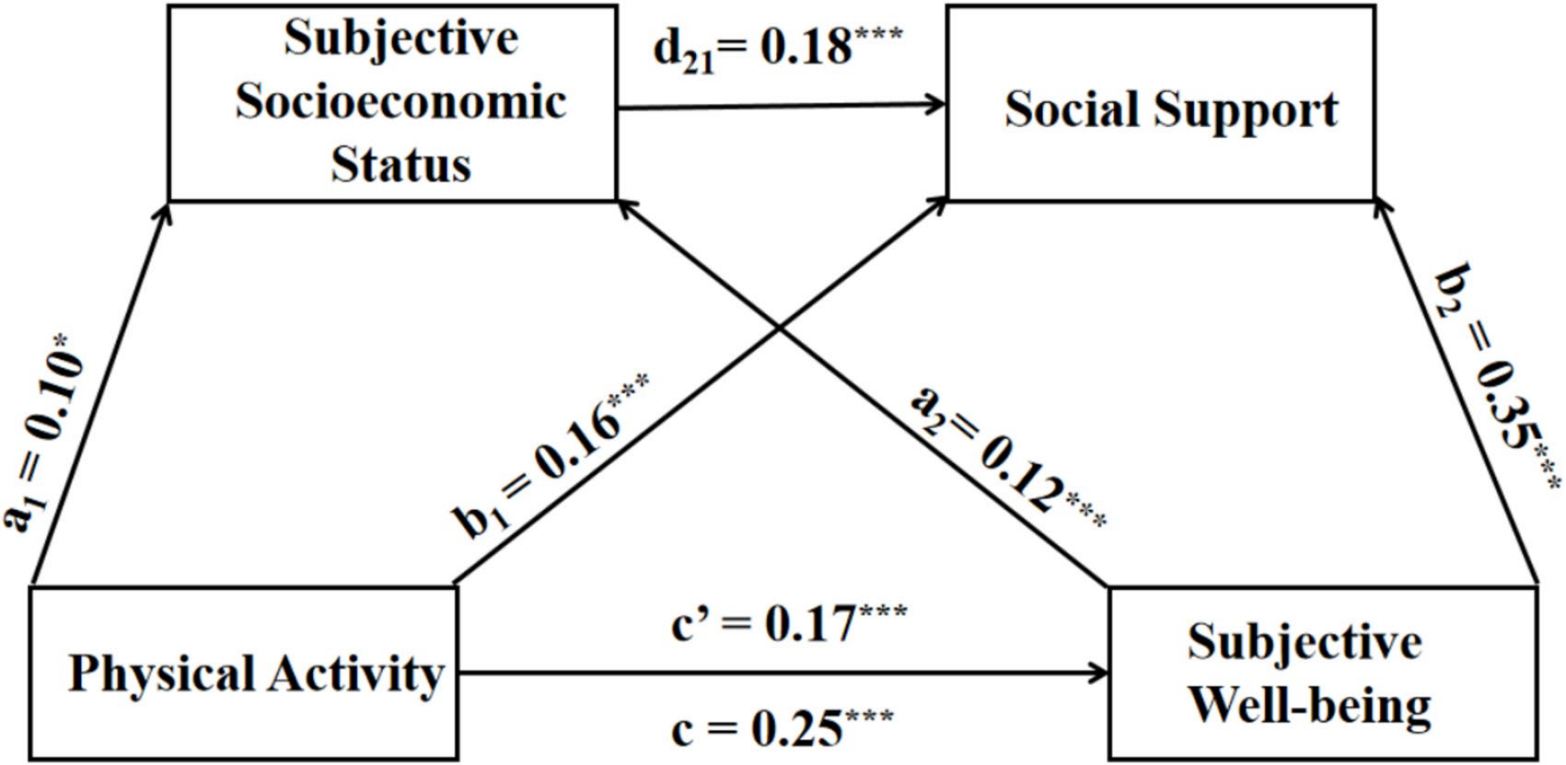
Chain mediation model of young adults’ physical activity and subjective wellbeing.

After accounting for the variables of age and gender, we conducted a mediation analysis using PROCESS 3.3 (Model 6) developed by Hayes. In this examination, physical activity was specified as the independent variable, subjective wellbeing as the dependent variable, and subjective socioeconomic status and social support were identified as the mediating variables. The results are summarized in Table 3. All regression coefficients (*β*) and 95% confidence intervals (CIs) are indeed based on standardized data. Engagement in physical activity can directly and notably foretell levels of subjective wellbeing (*β* = 0.25, *t* = 6.18, *p* < 0.001), 95%CI = [0.090, 0.174] (Model 1). Upon incorporating the two mediators: subjective socioeconomic status and social support, the findings demonstrated that physical activity could notably and positively predict subjective socioeconomic status (*β* = 0.10, *t* = 4.21, *p* < 0.001), 95%CI = [0.002, 0.015] (Model 2) (see Figure 2, a_1_ path). As showed in Model 3, our results indicate that physical activity serves not only as a critical component of physical health but also significantly and positively predicts social support (*β* = 0.16, *t* = 4.05, *p* < 0.001), 95%CI = [0.023, 0.067] (Model 3) (see Figure 2 b_1_ path), and subjective socioeconomic status was positively associated with social support (*β* = 0.17, *t* = 4.50, *p* < 0.001), 95%CI = [0.326, 0.833] (Model 3) (see Figure 2, d_21_ path). Furthermore, subjective socioeconomic status (*β* = 0.12, *t* = 3.39, *p* < 0.001), 95%CI = [0.332, 1.247] (Model 4) (see Figure 2, a_2_ path), and social support (*β* = 0.35, *t* = 9.56, *p* < 0.001), 95%CI = [0.541, 0.820] (Model 4) (see Figure 2, b_2_ path) positively predicted subjective wellbeing, meanwhile, the direct impact of physical activity on subjective wellbeing proved to be statistically significant (Model 4), (*β* = 0.17, *t* = 4.60, *p* < 0.001), 95%CI = [0.053, 0.131] (see Figure 2, c’ path).

**Table 3:** Multiple regression of the mediation effect.

Predictors	Model 1 (subjective wellbeing)		Model 2 (subjective socioeconomic status)		Model 3 (social support)		Model 4 (subjective wellbeing)	
	*β*	*t*	*β*	*t*	*β*	*t*	*β*	*t*
Gender	−0.07	−1.80	−0.02	−0.58	0.10	2.59	−0.10^***^	−2.82
Age	0.03	0.65	0.01	0.17	−0.06	−1.57	0.05	1.27
Physical activity	0.25^***^	6.18	0.10^**^	2.41	0.16^***^	4.05	0.17^***^	4.60
Subjective socioeconomic status					0.18^***^	4.50	0.12^***^	3.39
Social support							0.35^***^	9.56
*R*	0.277		0.11		0.26		0.47	
*R* ^2^	0.077		0.01		0.07		0.22	

*p < 0.05, **p < 0.01, and ***p < 0.001.

The mediating effects of subjective socioeconomic status and social support on the relationship between physical activity and subjective wellbeing were analyzed using the bias-corrected non-parametric percentile Bootstrap method. This approach involved generating 5,000 bootstrap samples to estimate the confidence interval at the 95% level. Results of the mediation effect test are presented in Table 4 and Figure 2. Specifically, subjective socioeconomic status plays a partial mediating role between physical exercise and subjective wellbeing, with the ratio of the mediating effect to the total effect being 4.93%. Social support exerts a partial mediating effect between physical exercise and subjective well-eing, and the ratio of this mediating effect to the total effect accounts for 23.12%. Subjective socioeconomic status and social support jointly form a partial serial mediating chain between physical exercise and subjective wellbeing, where the ratio of the serial mediating effect to the total effect reaches 2.43%. Thus, this finding suggests that subjective socioeconomic status and social support partially mediate the correlation between physical activity and subjective wellbeing.

**Table 4:** Decomposition of the effect of physical activity on subjective wellbeing.

Influence path	Normalized effect size	Boot SE	Boot LLCL Boot ULCI
Direct effect	0.0904	0.02	0.0510, 0.1297
Physical activity → subjective socioeconomic status → subjective wellbeing	0.0065	0.0039	0.0004, 0.0155
Physical activity → social support → subjective wellbeing	0.0305	0.0089	0.0143, 0.0493
Physical activity →subjective socioeconomic status → social support → subjective wellbeing	0.0032	0.0018	0.0003, 0.0073
Total indirect effect	0.0402	0.0097	0.0224, 0.0607
Total effect	0.1319	0.0213	0.0900, 0.1737

## 4 Discussion

This research explored the interconnections between physical activity, subjective socioeconomic status, social support, and subjective wellbeing among young adults. The involvement of young adults in physical activity is positively associated with their subjective sense of wellbeing, and this relationship may be partially explained by their perceived socioeconomic status and the social support they obtain. Consistent with anticipations, the results indicated that physical activity, subjective socioeconomic status, and social support are all positively and significantly related to subjective wellbeing. This corroborates prior research, which has established that young adults who participate more regularly in physical activities are more likely to report greater subjective wellbeing than their counterparts who are less physically active (Buecker et al., 2021; Liao C. et al., 2023; Liao T. et al., 2023; Mansfield et al., 2018; Zhang et al., 2022). Notably, even after controlling for variables such as age and gender, the study’s results underscored the significant predictive power of physical activity on young adults’ subjective wellbeing. This indicates that physical activity is a crucial extrinsic factor in the enhancement of subjective wellbeing.

Building on the findings, it was determined that young adults’ engagement in physical activity is positively associated with their subjective wellbeing, and subjective socioeconomic status and social support may function as mediating factors. Specifically, young adults who partake in higher levels of physical activity tend to possess a more elevated subjective socioeconomic status and receive greater social support, which may in turn be related to stronger subjective wellbeing. This aligns with Social Comparison Theory (Festinger, 1957), which posits that individuals with lower subjective socioeconomic status and social support—often engaging in less physical activity—tend to exhibit poorer health and lower subjective wellbeing. Moreover, the study’s outcomes are partially congruent with a contemporary investigation into the nexus between physical activity and subjective wellbeing among Chinese college students, highlighting that increased physical activity is related to higher subjective wellbeing through an enhanced level of social support (Liao C. et al., 2023; Liao T. et al., 2023). Additionally, the findings appear to endorse the Social Support Mediation Model (Kong et al., 2015, 2019; Kong and You, 2013; Yan et al., 2021). Our results extend the existing literature by elucidating that subjective socioeconomic status and social support could make significant contributions to the interplay between physical activity and subjective wellbeing within the general young adult demographic, employing a chain mediation model. This could further the understanding of the domain of physical activity and subjective wellbeing.

Significantly, the current study has demonstrated that subjective socioeconomic status and social support serve as sequential intermediaries in the link between young adults’ engagement in physical activity and their levels of subjective wellbeing. This finding supports the potential mediating role of both subjective socioeconomic status and social support within this context. That is, young adults with higher physical activity tended to have higher subjective socioeconomic status than those with lower physical activity, and further enhanced their social support, which may contribute to their subjective wellbeing. In the framework of this study, physical activity is related to young adults’ subjective wellbeing not solely through a direct association, but also via an indirect pathway that is facilitated by the intermediary roles of subjective socioeconomic status and social support. These factors interact to shape individuals’ wellbeing. Social support amplifies the health benefits of physical activity by fostering adherence and emotional reinforcement, while higher socioeconomic status directly enables greater physical activity participation through resource accessibility (e.g., gym memberships, safe exercise environments). Concurrently, physical activity generates social capital, such as new interpersonal connections and strengthened community ties, which in turn enhances social support networks. This reciprocal relationship forms a virtuous cycle: socioeconomic status facilitates physical activity, which cultivates social support, thereby boosting subjective wellbeing. This dynamic is rooted in the social ecological model, where individual behavior interacts with structural and relational factors to shape health outcomes. In addition, an increase in socioeconomic status may also enhance individuals’ ability to obtain social support, because individuals with higher socioeconomic status may have broader social networks and more social resources. These are in line with previous research. For instance, some research has shown that those who engage in more frequent physical activity tend to report a higher sense of subjective wellbeing (Buecker et al., 2021; Stathi et al., 2002; Wicker and Frick, 2017; Wiese et al., 2018; Yao et al., 2023; Zhang et al., 2022).

Furthermore, research has established a significant association between high subjective socioeconomic status and elevated social support (Bakchich et al., 2023; Hooker et al., 2020; Tinajero et al., 2015). This social support, in turn, serves as a key mediator of subjective wellbeing: studies show that individuals with stronger social support networks exhibit higher wellbeing scores (Chu et al., 2015; Nagy-Pénzes et al., 2020). The mediating role of social support is further supported by longitudinal data, which indicates that increases in social support over time are directly linked to improvements in subjective wellbeing, independent of initial socioeconomic status (Nagy-Pénzes et al., 2020). These results suggest that physical activity may be associated with subjective wellbeing through subjective socioeconomic status and support as mediators. This is in line with prior research indicating that subjective socioeconomic status is a key factor influencing social support (Han et al., 2021; Hooker et al., 2018).

Due to the cross-sectional design of this study, causal inferences cannot be drawn. It is also possible that individuals with higher subjective wellbeing or stronger social support are more likely to engage in physical activity.

### 4.1 Research limitations

Several limitations in this research need to be paid attention to. Firstly, as previously noted, young adulthood is a pivotal phase in human development, marked by substantial cognitive, emotional, and social transformations. The significance of understanding how physical activity relates to subjective wellbeing in this demographic is thus underscored. However, the research sample was strictly confined to young adults in China, which undermines the generalizability of these findings across cross-cultural and demographic contexts. Specifically, the absence of comparative data from non-Western societies, where social norms around physical activity and subjective wellbeing may differ systematically, and the lack of demographic diversity limit the validity of extrapolating these results to broader populations. Secondly, the cross-sectional design of this study inherently limits causal inference due to its inability to account for temporal precedence and confounders. Furthermore, our reliance on self-reported frequency to measure physical activity lacks granularity, as it does not distinguish between specific activity domains or capture daily variability. Additionally, the absence of anthropometric measures prevents us from accounting for potential physiological confounders that may moderate these relationships. Thirdly, the use of a convenience sample may have introduced selection bias. The demographic composition of the sample, particularly the gender ratio, might not be fully representative of the general student population. Future studies should adopt more rigorous sampling strategies, such as stratified random sampling, to ensure a balanced gender ratio and better representativeness of the broader student population. Fourthly, since this study relies on participants’ self-reported data, participants may exhibit “positive response bias” to align with general social expectations. Future studies could incorporate objective measurement methods to mitigate the impact of such bias. Fifthly, our assessment of physical activity relied primarily on frequency and did not capture specific domains or daily variability in activity patterns. Future studies should consider using objective measures to capture real-time fluctuations. Additionally, we did not collect anthropometric data, which could moderate the relationship between physical activity and subjective wellbeing. Future research integrating physiological markers would provide a more comprehensive view. Lastly, the measurement of physical activity only focuses on weekly exercise frequency and does not include indicators such as exercise duration or exercise intensity. This may fail to fully capture the complexity of physical activity and its potential associations with mediating variables.

In terms of practical applications, the findings of this study can provide insights for designing targeted interventions aimed at enhancing the subjective wellbeing of young adults in China. For example, educational institutions and community organizations can develop physical activity programs tailored to the characteristics of Chinese youth, integrating elements that promote positive social interactions and boost subjective socioeconomic status. These programs could potentially be adjusted and implemented in other cultural contexts after further validation. Regarding prospects, future research could expand the sample scope to include young adults from diverse cultural backgrounds, using cross-cultural comparative studies to explore the moderating role of culture in the relationship between physical activity, subjective socioeconomic status, social support, and subjective wellbeing. Additionally, integrating objective, real-time physiological monitoring techniques—such as wearable devices to track physical activity with high temporal resolution—and anthropometric assessments (e.g., BMI) can help establish more precise causal relationships. Moreover, Latent Growth Modeling (LGM), as a robust longitudinal analytical tool, should be adopted in long-term follow-up studies. It enables the tracking of dynamic changes in physical activity levels and the developmental trajectories of subjective wellbeing across different life stages of young adults, addressing the limitation of cross-sectional design in clarifying temporal precedence. This approach will provide more comprehensive and practical guidance for promoting youth mental health and overall wellbeing.

## 5 Conclusion

This research shows that physical activity is positively associated with subjective wellbeing among young adults, and this association is partially explained by the sequential mediating roles of subjective socioeconomic status and social support. The present study examined the relationship between physical activity and subjective wellbeing, aiming to deepen our understanding of the psychological factors and mechanisms that influence subjective wellbeing. This study extends previous findings by demonstrating that the link between physical activity and subjective wellbeing among young adults can be understood through a dual-mediational pathway involving subjective socioeconomic status and social support. These findings have important practical implications. In terms of intervention design, community programs that combine group exercise (to foster social support) with status-affirming activities (such as skills workshops) may offer potential value for improving wellbeing among young adults. In terms of practice implications, the results highlight the value of promoting physical activity, enhancing social support, and strengthening perceived social status in wellbeing promotion programs. Given the cross-sectional design and convenience sampling of this study, conclusions regarding causality and strong policy recommendations should be drawn with caution. Future longitudinal and experimental studies are needed to verify the causal directions and effectiveness of interventions.

## Data Availability

The raw data supporting the conclusions of this article will be made available by the authors, without undue reservation.
